# Homocysteine: Biochemistry, Molecular Biology and Role in Disease

**DOI:** 10.3390/biom11050737

**Published:** 2021-05-15

**Authors:** Anton Hermann, Guzel Sitdikova

**Affiliations:** 1 Department of Biosciences, University Salzburg, Hellbrunnerstr 34, A-5020 Salzburg, Austria; 2 Department of Human and Animal Physiology, Institute of Fundamental Medicine and Biology, Kazan Federal University, Kazan 420008, Russia

Homocysteine is a non-proteinogenic sulfhydryl-containing amino acid derived from methionine and is a homologue of cysteine. The concentration of homocysteine is regulated by two key pathways: remethylation back to methionine or transsulfuration to cysteine with the simultaneous production of hydrogen sulfide (H_2_S). Homocysteine levels can be increased by different conditions including genetic factors, diet, life style, several medications, etc. Elevated levels of homocysteine, called hyperhomocysteinemia (hHcy), are associated with a higher risk of neurovascular diseases, dementia, migraine, developmental impairments or epilepsy. The mechanisms underlying the neurotoxicity of homocysteine include oxidative stress, DNA damage, protein thiolation or protein homocysteinylation, triggering apoptosis and excitotoxicity. Recent data indicate that inflammation during hHcy comes along with increased levels of several cytokines and changes in DNA methylation.

The gasotransmitter H_2_S is implicated in the regulation of numerous physiological functions and possesses neuroprotective potential. Recent data indicate that the level of H_2_S decreases under hHcy conditions, which may mediate homocysteine-induced neurotoxicity.

The aim of this Special issue is to present recent findings of the pathologies associated with hHcy, mechanisms of homocysteine action and the protective role of H_2_S. The collection includes six original papers and three reviews comprising 49 authors who are experts in the field.

In his review, Brzezinski [1] summarizes findings on *S*-adenosyl-l-homocysteine Hydrolase (SAHase), which is a major regulator of cellular methylation reactions that occur in eukaryotic and prokaryotic organisms. SAHase activity is a significant source of homocysteine and adenosine. The author presents structural characteristics of the two principal domains of the SAHase subunit, which are based on the Rossmann fold. The study highlights similarities and differences in the spatial arrangements of both major domains. 

In their review Rizzo and Lagane [2] summarize data about the link between homocysteine and omega-3 polyunsaturated fatty acids, with a special focus on the meta-analyses of randomized clinical trials. The authors suggest that a synergic action between polyunsaturated fatty acids and B vitamins, if supplemented, may simultaneously play a key role in regulating several metabolic pathways and could be beneficial for individual health and healthcare policy.

hHcy which has been linked to different systemic and neurological diseases, is well known as a risk factor for systemic atherosclerosis and cardiovascular disease (CVD), and has been identified as a risk factor for several ocular disorders, such as diabetic retinopathy (DR) and age-related macular degeneration (AMD). Oxidative stress, endoplasmatic reticulum (ER) stress, inflammation, and epigenetic modifications have been suggested as possible mechanisms of hHcy-induced blood retinal barrier (BRB) dysfunction. More recently hHcy-induced brain inflammation was reported as a mechanism of blood-brain barrier (BBB) dysfunction and pathogenesis of Alzheimer’s disease (AD). The contribution by Tawfik et al. [3] focuses on the effects of hHcy on BRB and the controversial role of HHcy in the pathogenesis of aging neurological diseases such as DR, AMD, and AD, and it highlights the possible mechanisms caused by such deleterious effects of hHcy.

The original study by Elsherbiny et al. [4] demonstrates that inflammation is an underlying mechanism of hHcy-induced pathology in age-related diseases such as AMD, DR, and AD and that the elimination of excess Hcy or reduction of inflammation is a promising intervention for mitigating this kind of damage. 

In the paper by Kovalska et al. [5], a methionine (Met)-rich diet was used to produce hHCy, which was demonstrated to be a risk factor for atherosclerosis-associated diseases like stroke, dementia or Alzheimer’s disease. Their results suggest that the combination of the two risk factors (hHcy and ischemia-reperfusion insult (IRI) endorses and exacerbates rat hippocampal neurodegenerative processes in rats.

Homocysteine (HCY) combines distinct pharmacological properties as an agonist of *N*-methyl-d-aspartate receptors (NMDARs) and a reducing agent. In the paper by Sibarov et al. [6], the role of GluN2 Subunit-Dependent Redox Modulation of NMDA receptors was shown during severe hHCy. 

Ivanova et al. [7] provide evidence that protein kinase C (PKC) and protein kinase A (PKA) are involved in pathways of neuronal survival caused by ouabain in hHCy, which suggests the existence of different appropriate pharmacological treatments for hHCy and glutamate excitotoxicity.

Hydrogen sulfide has been implicated in cardiovascular protection through redox balance and vessel relaxation. The paper by Wijerathne et al. [8] emphasizes the role of the endogenous production of H_2_S in kidney ischemia-reperfusion injury and oxidative stress in the heart.

Finally, Yakovleva et al. [9] accentuate the protective role of H_2_S. Prenatal hyperhomocysteinemia (hHCy) was found to induce behavioral impairments and oxidative stress in brain tissue of rats, with a decreased expression of the H_2_S generating enzyme-cystathionine-beta synthase and concentrations of H_2_S in the brain. The administration of the H_2_S donor to females with hHcy during pregnancy prevented behavioral alterations and oxidative stress of their offspring. The acquisition of behavioral studies together with biochemical studies will improve our knowledge about homocysteine neurotoxicity and proposes H_2_S as a potential agent for the therapy of hHcy-associated disorders.

This Special Issue describes several pathological conditions associated with elevated levels of homocysteine, some further mechanisms of homocysteine action, and opens new possibilities for treatments. 
